# In vitro Antioxidant, Anti-inflammatory, Anti-metabolic Syndrome, Antimicrobial, and Anticancer Effect of Phenolic Acids Isolated from Fresh Lovage Leaves [*Levisticum officinale* Koch] Elicited with Jasmonic Acid and Yeast Extract

**DOI:** 10.3390/antiox9060554

**Published:** 2020-06-25

**Authors:** Anna Jakubczyk, Urszula Złotek, Urszula Szymanowska, Kamila Rybczyńska-Tkaczyk, Krystyna Jęderka, Sławomir Lewicki

**Affiliations:** 1 Department of Biochemistry and Food Chemistry, University of Life Sciences in Lublin, Skromna Str. 8, 20-704 Lublin, Poland; anna.jakubczyk@up.lublin.pl (A.J.); urszula.szymanowska@up.lublin.pl (U.S.); 2 Department of Environmental Microbiology, The University of Life Sciences in Lublin, Leszczyńskiego Street 7, 20-069 Lublin, Poland; kamila.rybczynska-tkaczyk@up.lublin.pl; 3 Department of Regenerative Medicine and Cell Biology, Military Institute of Hygiene and Epidemiology Kozielska 4, 01-163 Warsaw, Poland; krystyna.jederka@wihe.pl (K.J.); lewickis@gmail.com (S.L.)

**Keywords:** elicitation, lovage, antioxidant activities, anti-inflammatory potential, metabolic syndrome, antimicrobial potential, cytotoxic properties, phenolic acids, bioavailability in vitro

## Abstract

Lovage seedlings were elicited with jasmonic acid (JA) and yeast extract (YE) to induce the synthesis of biologically active compounds. A simulated digestion process was carried out to determine the potential bioavailability of phenolic acids. Buffer extracts were prepared for comparison. The ability to neutralize ABTS radicals was higher in all samples after the in vitro digestion, compared to that in the buffer extracts. However, the elicitation resulted in a significant increase only in the value of the reduction power of the potentially bioavailable fraction of phenolic acids. The effect of the elicitation on the activity of the potentially bioavailable fraction of phenolic acids towards the enzymes involved in the pathogenesis of the metabolic syndrome, i.e., ACE, lipase, amylase, and glucosidase, was analyzed as well. The in vitro digestion caused a significant increase in the ability to inhibit the activity of these enzymes; moreover, the inhibitory activity against alpha-amylase was revealed only after the digestion process. The potential anti-inflammatory effect of the analyzed extracts was defined as the ability to inhibit key pro-inflammatory enzymes, i.e., lipoxygenase and cyclooxygenase 2. The buffer extracts from the YE-elicited lovage inhibited the LOX and COX-2 activity more effectively than the extracts from the control plants. A significant increase in the anti-inflammatory and antimicrobial properties was noted after the simulated digestion.

## 1. Introduction

Numerous investigations have demonstrated the various biological activities of herbs, e.g., digestive stimulant, antioxidant, anti-inflammatory, antibacterial, or antiproliferative properties. Many studies have shown that the intake of some medicinal herbs may be associated with a lower incidence of some cardiovascular, cancer, or other chronic diseases [1,2].

Lovage is a plant from the *Apiaceae* family. It is described in the literature as a valuable component of the human diet due to its content of compounds with documented bioactive properties that are beneficial for human health. This herb is known for its culinary value and is widely used by both the condiment industry and by households as an ingredient of soups, stews, meat dishes, etc. However, the traditional use of lovage is not limited to cuisine. Its medicinal carminative, spasmolytic, and diuretic properties have been known for a long time. Moreover, a decoction extract made from the aerial parts of the plant has been described as an antiseptic agent for wounds [3]. The main groups of secondary metabolites contained in lovage are essential oils, polyphenols, coumarins, alkaloids, and polyacetylenes [4]. A big group of bioactive compounds present in lovage leaves comprises phenolic compounds represented by the following subgroups: phenolic acids, flavonoids, tannins, stilbenes, curcuminoids, coumarins, lignans, quinones, and others. Phenolic compounds have a diverse range of beneficial biological activities responsible for their potent effect on many chronic diseases. Their physiological and pharmacological functions are mainly associated with the well documented antioxidant and free radical scavenging properties and their function in the regulation of detoxifying enzymes [4,5].

Besides their strong antioxidant activities, natural phenolic compounds from herbs have anti-inflammatory properties. The mechanisms of the anti-inflammatory activity of polyphenols include the regulation of the production of pro-inflammatory factors, the modulation of the activity of inflammation-associated cells or genes, and the impact on the activity of enzymes involved in the metabolism of arachidonic acid, i.e., lipoxygenase and cyclooxygenase [6,7].

It is well documented that oxidative stress and chronic inflammation predispose the organism to some diseases, including cancer. One of the mechanisms of the protective action of phenolics against carcinogenesis is related to their free radical scavenging activity and inhibition of the formation of inflammatory mediators [8,9]. Phenolic acids are found ubiquitously and are well documented for many health protective effects like antioxidant, anti-inflammatory, antimicrobial or anticancer. The association of parent phenolic acids and their metabolites has been confirmed by in vitro and in vivo studies [10].

There is evidence that phenolic compounds can inhibit certain enzymes involved in the pathogenesis of diseases and thus contribute to the inhibition of metabolic syndrome (MS) symptoms, i.e., hypertension, obesity, hyperglycemia, or insulin resistance [10,11]. Therefore, intensive research should be carried out to demonstrate that these compounds can be regarded as food ingredients and that they cause less serious side effects than medicines.

Considering these facts, the search for methods to increase the content of phenolic compounds and/or other bioactive compounds in food of plant origin is still the subject of many studies. In the case of vegetables and herbs that can be consumed in the fresh unprocessed form, it is advisable to use such methods at the stage of cultivation. One of the methods improving plant resistance and inducing the production of secondary metabolites, which are plant bioactive compounds, is elicitation [12,13]. Elicitation is a method of inducing physiological changes in the plant by triggering signal pathways that are responsible for plant defense responses. It is a method for the natural induction of plant resistance mechanisms and the synthesis of phytochemical compounds, which is especially important in edible plants. Thus, elicitation can improve the pro-health quality of food of plant origin. Various methods of applying elicitors to the plant are known, and the most commonly used include soaking seeds in elicitor solutions, the exogenous spraying of plant leaves, or using a hydroponic system [14,15]. There are many studies of the impact of elicitation on the content and bioactivity of plant phytochemicals, but they are predominantly focused on “chemical extracts” [14,15,16], and there is limited information about the potential bioaccessibility of these compounds from elicited plants [17,18]. 

Comprehensive knowledge of some variables of the bioavailability of polyphenols is still needed, since the effect of the dietary compounds is not fully understood. The bioavailability and action of phenolic compounds on the health of consumers is determined by both the interaction with other components of the food matrix, e.g., proteins, carbohydrates, or lipids, and the impact of the digestive tract environment. Digestive enzymes can induce the transformation of some phenolics, liberation thereof from the food matrix, and conversion in the digestion conditions [19,20].

In the present study, we investigated the impact of elicitation of lovage with jasmonic acid and yeast extract on the content and bioactivity (determined as antioxidative, anti-inflammatory, antimicrobial, and antiproliferative potential as well as the inhibition of enzymes involved in MS symptoms) of potentially bioavailable fractions of phenolic acids.

## 2. Materials and Methods

### 2.1. Chemicals

Jasmonic acid, yeast extract, ferrozine (3-(2-pyridyl)-5,6-bis-(4-phenyl-sulfonic acid)-1,2,4-triazine), 2,20-azino-bis (3-ethylbenzothiazoline-6-sulphonic acid) (ABTS), α-amylase, pancreatin, pepsin, lipase, α-glucosidase, bile extract, lipoxygenase, linoleic acid, angiotensin converting enzyme, o-phthalaldehyde, *p*-nitrophenyl acetate, dimethyl sulfoxide, 3,5-dinitrosalicylic acid, *p*-nitrophenol, protocatechuic acid, syringic acid, vanillic acid, sinapic acid, salicylic acid, caffeic acid, and hydroxybenzoic acid, cyclohexamide, resazurin were purchased from Sigma-Aldrich (Poznan, Poland). The COX Activity Assay kit was purchased from Cayman Chemical company (Ann Arbor, MI, USA). Penicillin and streptomycin were purchased from Life Technologies, Warsaw, Poland. Mueller–Hinton broth, and Mueller–Hinton agar were also obtained (Biomaxima, Lublin, Poland). All other chemicals were of analytical grade.

### 2.2. Plant Material

Fresh lovage leaves (*Levisticum officinale* Koch. cv. Elsbetha) elicited with 10 µM jasmonic acid (JA) and 0.1% yeast extract (YE) during growth in controlled conditions were the study material. The growth conditions and method of elicitation were described in our previous manuscript [21]. Lovage seeds (*Levisticum officinale* Koch. cv. Elsbetha) were obtained from the Enza Zaden Company (Enkhuizen, the Netherlands). The plants were grown from seeds in controlled phytotron conditions (SANYO MLR-350H) at 25/18 °C, photoperiod 16/8 h day/night, with photosynthetic photon flux density (PPFD) at a plant level of 500–700 µmol m^−2^s^−1^ and 75% relative humidity, which prevented the contamination of the crop by other accidental species. A voucher specimen of the plant material (no 20.10.2019) was deposited in the Department of Biochemistry and Food Chemistry, University of Life Sciences in Lublin, Skromna Str. 8. Twenty-one-day-old plants were sprayed with a water solution of the tested elicitors (1.5 mL per plant). The concentrations of the elicitors were selected based on the previous study [21], which indicated that the 0.1% yeast extract (YE) and 10 µM jasmonic acid (JA) proved to be the most effective concentrations of the elicitors for lovage plants. Fresh leaves were harvested, frozen, and stored until further analysis.

### 2.3. Preparation of Extracts

#### 2.3.1. Buffer Extracts

For the preparation of buffer extracts, frozen leaf tissue (1 g) was homogenized, extracted for 30 min with 20 mL of PBS buffer (phosphate buffered saline, pH 7.4), and centrifuged at 9000× *g* for 20 min. Then, the residues were extracted again with 20 mL of PBS buffer. The supernatants were combined and adjusted to a final volume of 50 mL of with the PBS buffer.

#### 2.3.2. In Vitro Digestion

In vitro digestion was performed as described previously by Gawlik-Dziki et al. [22]. The frozen leaf tissue (1 g) was homogenized in a Stomacher Laboratory Blender for 1 min to simulate mastication in the presence of 5 mL of a simulated saliva solution containing 7 mM NaHCO_3_, 0.35 mM NaCl (pH 6.75), and α-amylase (E.C. 3.2.1.1., 200 U per mL of enzyme activity). Subsequently, the mixture was stirred for 10 min at 37 °C in darkness. For gastric digestion, the solution was adjusted to pH 2.5 with 1 M HCl and 15 mL of 300 U/mL of pepsin (from porcine stomach mucosa, pepsin A, EC 3.4.23.1) in 0.03 mol/L NaCl, pH 1.2, were added. The reaction was carried out for 60 min. at 37 °C. Then, the solution was adjusted to pH 7 with 1 M NaOH and 15 mL of a mixture of a 0.7% solution of pancreatin and a 2.5% solution of bile extract were added. The incubation was carried out for 120 min. at 37 °C in darkness. Thereafter, the samples were centrifuged and the supernatants (gastrointestinally digested extracts; GD-extracts) were used for further analysis.

### 2.4. Analysis of Phenolic Compounds

#### HPLC Analysis

Quantitative–qualitative analyses of the phenolic acid extracts were performed using a varianprostar HPLC system separation module (Varian, Palo Alto, CA, USA) equipped with a Varian ChromSpher C18 reverse-phase column (250 mm × 4.6 mm) and a ProStar diode array detector. The column thermostat was set at 40 °C. All analyses were carried out with 4.5% acetic acid (solvent A) and 50% acetonitrile (solvent B) mobile phases at a flow rate of 0.8 mL min^−1^. At the end of the gradient, the column was washed with 50% acetonitrile and equilibrated to the initial condition for 10 min. Quantitative determinations were conducted with the calculation of an external standard, using calibration curves of the standards (phenolic acid standards used in the estimation: protocatechuic acid, syringic acid, vanillic acid, sinapic acid, salicylic acid, caffeic acid, and hydroxybenzoic acid). Detection was performed at 270 and 370 nm. Phenolic acids present in the sample were identified by comparing the retention times and the UV–visible absorption spectra with those of the standard compounds. The quantitative analysis of phenolic acids was based on calibration curves, which were plotted separately for each phenolic acid standard in a concentration range of 2.5–100 µg/mL. The phenolics were expressed as micrograms per gram fresh weight (FW) [23].

### 2.5. Antioxidant Activities

#### 2.5.1. Free Radical Scavenging Assay

Free radical scavenging activity was determined using 2,2′-azino-bis[3-ethylbenzothiazoline-6-sulphonic acid (ABTS^•+^) as a source of free radicals, as in Re et al. [24]. The antioxidant activity was expressed as μmol of Trolox per gram of fresh weight (FW) (TEAC, Trolox equivalent antioxidant activity).

#### 2.5.2. Ferric Reducing Antioxidant Power

Ferric reducing antioxidant power (RP) was determined according to the methods described by Oyaizu [25]. The reducing power was expressed as a Trolox equivalent (TE) in µmol of Trolox per gram of fresh weight (FW).

#### 2.5.3. Chelating Power

Chelating power (CHP) was determined using the method developed by Guo et al. [26]. The percentage of inhibition of the ferrozine-Fe^2+^ complex formation was calculated using the formula:

% inhibition = [1 − A_A_/A_C_] × 100

where:

A_C_—absorbance of the control (the solvent instead of the extract), A_A_—absorbance of the sample.

The chelating power was expressed as an EDTA (Ethylenediaminetetraacetic acid)(equivalent in µg EDTA per g of fresh weight (FW).

### 2.6. Determination of Potential Anti-Inflammatory Properties

#### 2.6.1. Lipoxygenase Inhibitory Activity

To analyze the effect of the different concentrations of the control and the elicited lovage extracts (PBS and obtained after the simulated digestion) on lipoxygenase activity, the method described by Szymanowska et al. [27] adapted to the BioTek Microplate Reader was used. Based on the relationship between the concentration of the analyzed extract and the degree of inhibition of LOX activity, a curve was drawn from which the EC_50_ value, i.e., an extract concentration (mg FW/mL) providing 50% inhibition, was determined.

#### 2.6.2. Cyclooxygenase-2 Inhibitory Activity

A COX Activity Assay kit from Cayman Chemical Company was used to determine the inhibition of cyclooxygenase-2 activity by the analyzed extracts. The determination was carried out according to the procedure specified by the manufacturer of the kit. Based on the degree of inhibition of COX-2 activity by various concentrations of the analyzed extracts, the EC_50_ value, i.e., a concentration (mg FW/mL) providing 50% enzyme inhibition, was determined.

### 2.7. Metabolic Syndrome Enzyme Inhibitory Activity Assay

#### 2.7.1. Angiotensin Converting Enzyme (ACE) Inhibitory Assay

The ACE inhibitory activity was measured with the spectrophotometric method with N-Hippuryl-l-histidyl-l-leucine (HHL) as a substrate using BioTek Microplate Readers [28].

#### 2.7.2. Lipase Inhibitory Activity Assay

Lipase activity was measured with p-nitrophenyl acetate (pNPA) as a substrate according to the method described by Wang et al. [29] with modifications developed by Złotek et al. [30].

#### 2.7.3. α-Amylase Inhibitory Assay

The α-amylase inhibitory activity (αAI) of the protein hydrolyzates and the peptide fractions was measured according to the method described by Świeca et al. [31].

#### 2.7.4. α-Glucosidase Inhibitory Assay (αGIA)

αGIA was measured with the method described by Jakubczyk et al. [32] using BioTek Microplate Readers.

### 2.8. Antimicrobial Properties

The samples were tested against bacteria: *Escherichia coli* American Type Culture Collection (ATCC) 25922, *Staphylococcus aureus* ATCC 29737, *Listeria monocytogenes* ATCC BBA-2660, *Bacillus cereus* ATCC 14579, and *Salmonella enteritidis* ATCC 4931 and yeast *Candida albicans* ATCC 90028. The strains were obtained from the American Type Culture Collection (ATCC, distributors: LGC Standards, Łomianki, Poland) and stored at 4 °C. All strains were cultured at 37 °C on nutrient broth (NB) medium.

#### 2.8.1. Determination of the Minimum Inhibitory Concentration (MIC) and Minimum Lethal Concentrations (MLC)

Serial twofold dilutions of each sample were made with Mueller–Hinton broth (MHB) to yield final concentrations ranging from 5 to 0.312 mg mL^−1^ for the studied buffer (PBS) extracts and GD-extracts and placed into 96-well plates. Then, 100 μL of bacterial (10^8^ CFU mL^−1^) or yeast (10^5^ CFU mL^−1^) cultures were added. The wells with MHB or bacterial cultures served as the negative and positive control, respectively. In the bacteria cultures, penicillin G and streptomycin were used as a positive control in a concentration ranging between 250 and 0.24 µg mL^−1^. In the case of *C. albicans* as a positive control, cyclohexamide (500–0.48 µg mL^−1^) was used. Minimum lethal concentrations (MLC) for the bacteria and yeast were determined after broth microdilution by subculturing the samples from the wells that showed no microbial growth onto the surface of a Mueller–Hinton agar (MHA) medium. The plates were incubated at 37 °C for 18 h. The MLC was defined as the lowest concentration of the antimicrobial agent needed to kill 99.9% of the final inoculum after 18 h of incubation.

#### 2.8.2. Estimation of the Biotoxicity of the Studied Samples Using Resazurin Reduction Assays

Resazurin reduction assays were performed to estimate the biotoxicity against the bacteria and yeast. After MIC estimation, 20 μL of a 60 μM resazurin solution in PBS buffer were added to each well. The assay was based on the detection of cell metabolic activity. Resazurin (7-hydroxy-3H-phenoxazin-3-one 10-oxide) enters the cell in an oxidized form (blue), where it is converted into a reduced form, i.e., resorufin (pink), and indicates the reduction capability of cells, which reflects the mitochondrial function and cell viability and shows time- and concentration-dependent cell growth inhibition [33]. After incubation (2 h, 37 °C), the cell viability was monitored by measuring the absorbance at 570 nm (reduced) and 600 nm (oxidized) and calculating bacterial viability (in %) against the control (bacteria or yeast grown without the samples).

### 2.9. Anticancer Properties

The studies were performed on two cancer cell lines: gastric epithelial cell line NCI-N87 (ATCC^®^ CRL5822™) and prostate cancer line VCaP (ATCC^®^ CRL-2876™). Healthy prostate epithelial cells HPrECs (ATCC^®^ PCS-440-010™) were used as a control. All cell lines were purchased from the American Type Culture Collection (ATCC), University Boulevard, Manassas, VI, USA.

#### 2.9.1. Cell Culture

The NCI-N87 cells were cultured in RPMI-1640 with the addition of 10% FBS and antibiotics (50 U/mL of penicillin and 50 µg/mL of streptomycin—both from Life Technologies, Warsaw, Poland). The VCaP cells were cultured in DMEM with the addition of 10% FBS and antibiotics (50 U/mL of penicillin and 50 µg/mL of streptomycin). HPrECs were maintained in a dedicated medium consisting of a basic medium (Prostate Epithelial Cell Basal Medium, ATCC, USA) and supplements (Prostate Epithelial Cell Growth Kit, ATCC, USA) without the addition of FBS but with the addition of antibiotics (50 U/mL of penicillin and 50 µg/ml of streptomycin, Life Technologies, Warsaw, Poland). The cells were cultured in standard conditions (37 °C, 95% humidity, 5% CO_2_) in compliance with aseptic principles. The cells for the experiments (from 4 to 10 passages) were grown in continuous cultures and passaged with the use of 0.25% trypsin after reaching 80–90% confluence. After thawing from the cell bank, the cells were passaged twice before the experiments. After trypsinization, the cells were centrifuged at 300× *g* for 5 min., and the cell pellets were resuspended in 1 mL of fresh media and counted (ADAM MC Automated Mammalian Cell Counter, Digital Bio, Seoul, Korea). Then, the assessment of cytotoxicity (MTT and NR assays, both from Sigma Aldrich, Poznan, Poland), cell viability, and cell cycle analysis were performed.

#### 2.9.2. MTT and NR Tests

The assay was performed as described previously [34]. After trypsinization (0.05% trypsin, 3–7 min, 37 °C), the cells were seeded onto a 96-cell culture plate at the following concentrations: (a) gastric cancer 2 × 10^3^ cells/well; (b) prostate cancer 4 × 10^3^ cells/well; (c) healthy prostate 5 × 10^3^ cells/well. After 24 h, the cells were washed with the PBS solution and the appropriate culture medium and the samples were added. The lovage leaf extracts were added at concentrations of 0 (control), 0.1, 1, 5, 10, and 20 mg /mL. After 24-, 48-, and 72-h incubation with the extracts, proliferation tests (MTT, NR) were performed. The intensity of the color obtained was determined using a Fluostar Omega microplate reader (FLUOstar Omega, BMG Labtech, Ortenberg, Germany). The absorbance was measured at 570 nm for the MTT test and 540 nm for the NR assay with the background cutoff at 690 nm. The results are presented as the mean ± SD of 3 independent experiments (n = 9).

#### 2.9.3. Cell Viability and Type of Cell Death

The study was conducted according to the procedure described by Leśniak et al. [35]. After trypsinization (0.05% trypsin, 3-7 min, 37 °C), the cells were seeded onto a 48-cell culture plate at the following concentrations: (a) gastric cancer 2 × 10^4^ cells/well; (b) prostate cancer 4 × 10^4^ cells/well; (c) healthy prostate 5 × 10^4^ cells/well. After 24 h, the cells were washed with the PBS solution, and the appropriate culture medium with the tested lovage leaf extracts was added at the concentrations of 0.1 and 20 mg/mL. After 24-h floating, the cells were collected and adherent cells were trypsinized (0.05% trypsin, 3 min, 37 °C). After trypsinization, all the cells (floating and adherent) were mixed and centrifuged (300× *g*, 5 min). They were resuspended in 100 uL of PBS; then, 4.5 μM APC conjugated V (BD Bioscience, Warsaw, Poland) and 8 µL/sample of propidium iodide (PI, BD Bioscience, Warsaw, Poland) were added. The cells were incubated for 15 min. at 4°C and analyzed immediately by flow cytometry (FACS Calibur, BD, San Jose, CA, USA). The percentages of live, apoptotic, and necrotic cells were analyzed using a flow cytometer; 10,000 cells stopped the acquisition. The results were analyzed by CellQuest Pro Software (BD, San Jose, CA, USA) and are presented as mean SD of 3 independent experiments (n = 6).

#### 2.9.4. Cell Cycle

After trypsinization (0.05% trypsin, 3–7 min, 37 °C), the cells were seeded onto a 48-cell culture plate at the following concentrations: (a) gastric cancer 1 × 10^5^ cells/well; (b) prostate cancer 1.5 × 10^5^ cells/well; (c) healthy prostate 2 × 10^5^ cells/well. After 24 h, the cells were washed with the PBS solution, and an appropriate culture medium with the studied lovage leaf extracts was added at the concentrations of 0.1 and 20 mg/mL. After 24 h, the cells were trypsinized (0.05% trypsin, 3 min, 37 °C), centrifuged (300× *g*, 5 min), washed twice with the PBS solution, and centrifuged again (300× *g*, 5 min). The cell pellet was then resuspended in 1 ml of cold 70% ethanol/PBS solution (4 °C) and left in a freezer (−20 °C) for the permeabilization of the cell membrane until the analysis (min. one day, max. 30 days). On the analysis day, the cells were centrifuged (300× *g*, 5 min) and suspended in 300 μL of stain buffer (PI/RNase Staining Buffer, BD Warsaw, Poland). Then, propidium iodide (PI, Bioscene Warsaw, Poland) was added for 15 min at room temperature. The cell cycle analysis was performed using a flow cytometer (FACS Calibur, BD, San Jose, CA, USA) (25,000 cells stopped the acquisition) and calculated using FCS Express program (De Novo Software, Pasadena, CA, USA). The results are presented as mean ± SD of 3 independent experiments (n = 6).

### 2.10. Statistical Analysis

All the determinations were performed in triplicate. Statistical analysis was performed using STATISTICA 7.0 software for a mean comparison in ANOVA with post-hoc Tukey’s HSD (honestly significant difference) test at the significance level *p* < 0.05.

Data obtained from the experiment on cancer cells were checked for the normality of distribution (Shapiro–Wilk test). The level of statistical significance was calculated: in the case of normal distribution, one-way ANOVA with Bonferroni correction and Student’s t test were used; in another case, non-parametric one-way ANOVA with correction Kruskal–Wallis and Mann–Whitney tests were employed. The data were analyzed using the GraphPad Prism program (version 5, GraphPad Software, Inc., La Jolla, CA, USA) at a significance level *p* < 0.05.

## 3. Results

### 3.1. Phenolic Acid Analysis

The qualitative and quantitative analysis of phenolic acids in the PBS and in vitro digested samples of the control and elicited lovage leaves are summarized in Table 1. The elicitation with JA caused an increase in the content of some compounds in the case of the PBS extracts, i.e., protocatechuic acid, syringic acid, vanillic acid, sinapic acid, salicylic acid, and caffeic acid, whose content increased 0.58-fold, 1.48-fold, 0.82-fold, 1.15-fold, over 5-fold, and over 3-fold, respectively (Table 1). Similarly, in the case of the GD samples, a higher concentration of protocatechuic acid and hydroxybenzoic acid was noted in the samples prepared from the jasmonic acid-elicited lovage. The YE elicitation resulted in an increase in the sinapic acid content in the PBS extract and hydroxybenzoic acid in the case of the GD samples—Table 1. There was no clear tendency in the influence of the simulated digestion of lovage leaves on the concentration of phenolic compounds. The simulated in vitro digestion resulted in an increase in the content of hydroxybenzoic acid (compared to the PBS extracts), while an opposite trend was observed in the case of e.g., protocatechuic acid or sinapic acid (Table 1).

### 3.2. Antioxidant Activities

The analysis of the elicitation effect on the antioxidant activity of the potentially bioavailable phenolic compounds contained in lovage showed an increased activity in the in vitro digested samples only in the case of the RP elicitation. The activity increased by 45.25% and 26.5% in the JA- and YE-elicited samples, respectively (Table 2). It should also be noted that the simulated digestion resulted in an increase in the antiradical activity (in all samples) and reducing power (in the case of the JA- and YE-elicited samples). In contrast, the in vitro digestion resulted in the reduction of the chelating capacity in all the tested samples—Table 2.

### 3.3. Potential Anti-Inflammatory Properties

**Figure 1:**
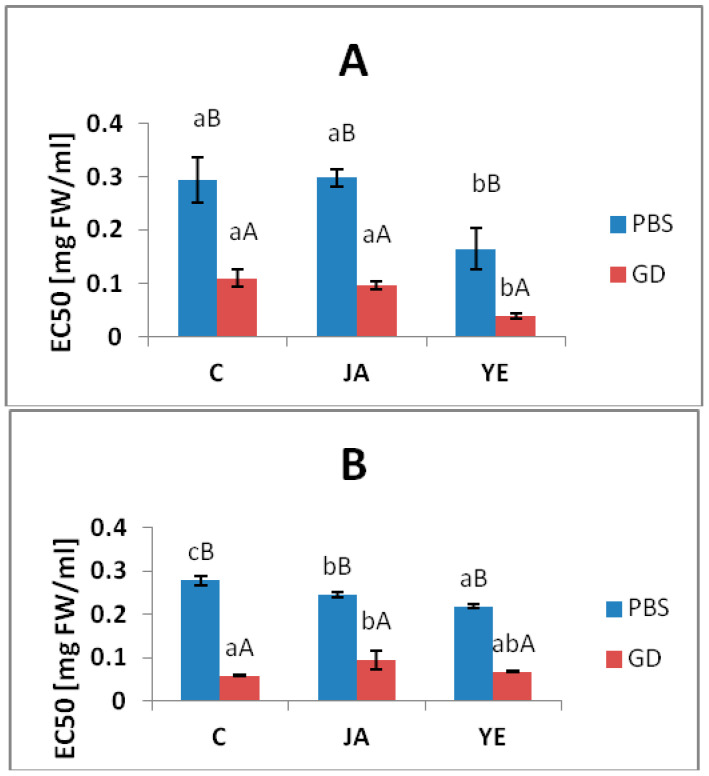
Lipoxygenase (**A**) and cyclooxygenase-2 (**B**) inhibitory activity in the control, elicited lovage buffer (PBS) extracts, and gastrointestinally digested (GD) extracts. C—extracts from the control plants; JA—extracts from the plants elicited with a solution of 10 µM jasmonic acid; YE—extracts from the plants elicited with 0.1% yeast extract. The averages with different capital letters show significant differences between the PBS and GD samples within one elicitation variant, and small letters denote significant differences between the elicitation variants for the PBS or GD samples (*p* < 0.05).

As shown in Figure 1, the samples inhibited the enzymes involved in inflammation, e.g., lipoxygenase and cyclooxygenase 2. The PBS-extractable phenolic compounds from the YE-elicited lovage showed a statistically significantly higher ability to inhibit LOX and COX 2 activity (the EC_50_ values were 0.16 ± 0.038 and 0.22 ± 0.07 mg FW/mL for LOX and COX2, respectively) relative to the control (EC_50_ = 0.29 ± 0.04 and 0.28 ± 0.05 mg FW/ml for LOX and COX2, respectively). The PBS extract from the JA-elicited lovage exhibited a higher ability to inhibit only COX2 activity: the COX2 inhibition potential increased by 25% in relation to the control (Figure 1b). It should be noted that the GD-extracts were characterized by a significantly higher ability to inhibit LOX and COX2 in comparison to the PBS extract, but only the GD-extract from the YE-elicited lovage exhibited a statistically significantly higher ability to inhibit LOX (ca. 65%) than the control (Figure 1).

### 3.4. Inhibitory Activity against Metabolic Syndrome Enzymes

The samples were checked as the potential inhibitors of enzymes involved in metabolic syndrome pathogenesis. This potential was determined by measuring the ACE, lipase, α-amylase and α-glucosidase inhibitory activity and expressed as EC_50_ values, as shown in Table 3.

In the case of the PBS-extractable phenolic compounds, the extracts from the JA- and YE-elicited lovage were characterized by a higher ability to inhibit ACE and lipase in comparison to the control lovage. Among the PBS extracts, the lowest EC_50_ for ACE inhibition was observed for the YE-PBS sample (EC_50_ = 97.68 ± 8.83 mg FW/mL), whereas the highest lipase inhibitory ability was exhibited by the JA-PBS extract (EC_50_ = 1.15 ± 0.05 mg FW/mL)—Table 3. It should also be noted that the buffer-extractable phenolic compounds from the control and elicited lovage leaves did not inhibit α-amylase, but this activity was observed in the in vitro digested samples—Table 3.

As in the case of the ability to inhibit pro-oxidative enzymes, the in vitro digestion significantly increased the ability of all the studied samples (control and elicited) to inhibit the activity of the enzymes involved in the pathogenesis of the metabolic syndrome—Table 3. Surprisingly, the elicitation did not have a positive effect on the potential of the bioavailable phenolic compounds (GD-extracts) from the fresh lovage leaves to inhibit these enzymes. Only the GD extract from the YE-elicited lovage exhibited a higher ability to inhibit α-amylase (ca. 15.3%) than the control (Table 3).

### 3.5. Antimicrobial Properties

The antimicrobial properties of the samples were tested against the bacteria: Escherichia coli ATCC 25922, Staphylococcus aureus ATCC 29737, Listeria monocytogenes ATCC BBA-2660, Bacillus cereus ATCC 14579, and Salmonella enteritidis ATCC 4931 and yeast Candida albicans ATCC 90028. The results showed that all GD samples had certain antimicrobial activity against all these microorganisms. No antimicrobial properties were detected in the case of the PBS samples (Table 4). The MIC values for the tested bacterial strains in the presence of the GD samples were in the range of 1.25–5.0 mg/mL (Table 4). The best antimicrobial properties of the in vitro digested samples were noted in the case of *B. cereus* and yeast with MIC = 1.25 and 2.0 mg/mL, respectively (Table 4). The GD samples exerted a lethal effect against *B. cereus* ATCC 14579 and C. albicans ATCC 90028 (MLC = 2.5–5.0 mg/mL) (Table 4).

**Figure 2:**
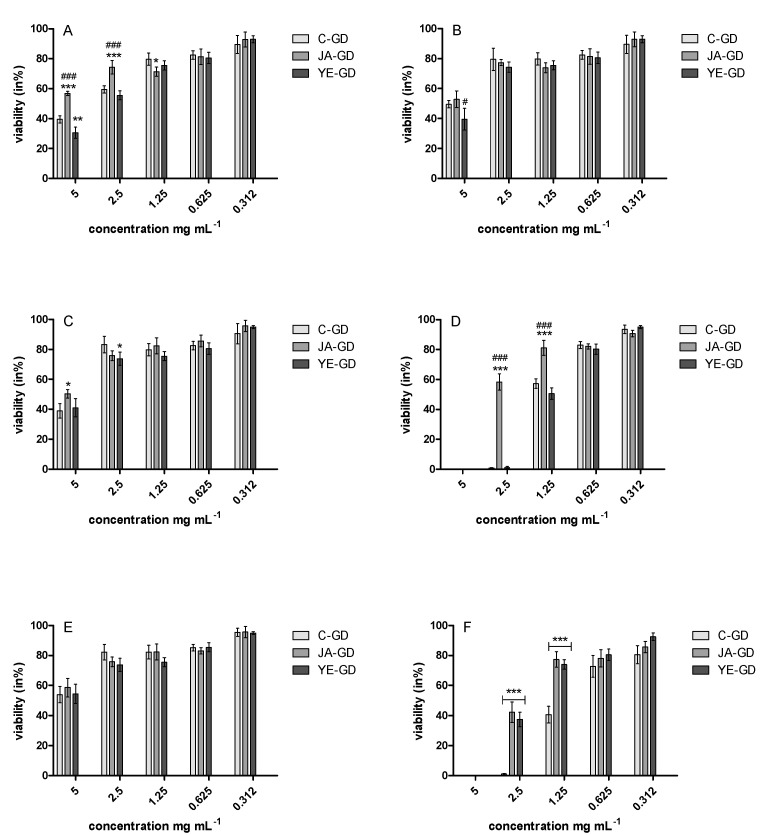
Viability (in %) of **E. coli** ATCC 25922 (**A**), *S. aureus* ATCC 29737 (**B**), *S. enteritidis* ATCC 4931 (**C**), *B. cereus* ATCC 14579 (**D**), *L. monocytogenes* ATCC BBA-2660 (**E**), and yeast *C. albicans* ATCC 90028 (**F**) against the gastrointestinally digested samples. C-GD—gastrointestinally digested extracts from the control plants; JA-GD—gastrointestinally digested extracts from the plants elicited with a solution of 10 µM jasmonic acid; YE-GD—gastrointestinally digested extracts from the plants elicited with 0.1% yeast extract.

This result was also confirmed by the resazurin reduction assay, which showed different rates of resazurin reduction by the tested microorganisms. In the case of the GD samples, the highest growth inhibition was observed for *B. cereus* ATCC 14579 and C. albicans ATCC 90028 (Figure 2). However, the elicitation did not significantly affect these properties—the YE-GD samples were more effective only in the case of *E. coli* (Figure 2A).

### 3.6. Anticancer Properties

**Figure 3:**
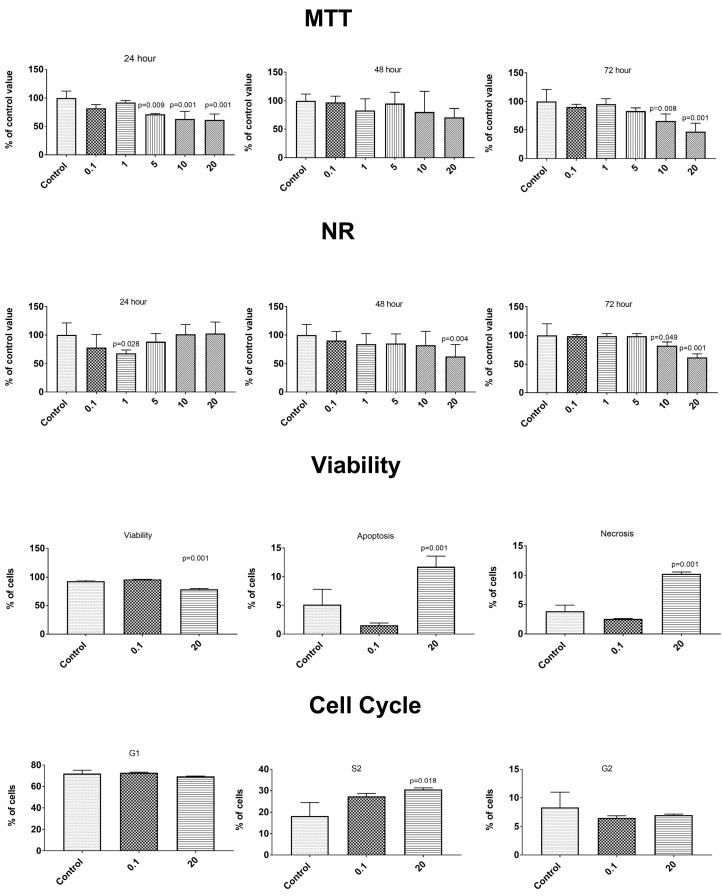
Impact of C-GD (sample after the in vitro digestion of the control lovage leaves) on cytotoxicity (MTT and NR method), type of cell death (Annex V and propidium iodide (PI) test), and the phases of the cell cycle in the healthy prostate epithelial cell (HPrEC) ATCC^®^ PCS-440-010 ™ cell line.

**Figure 4:**
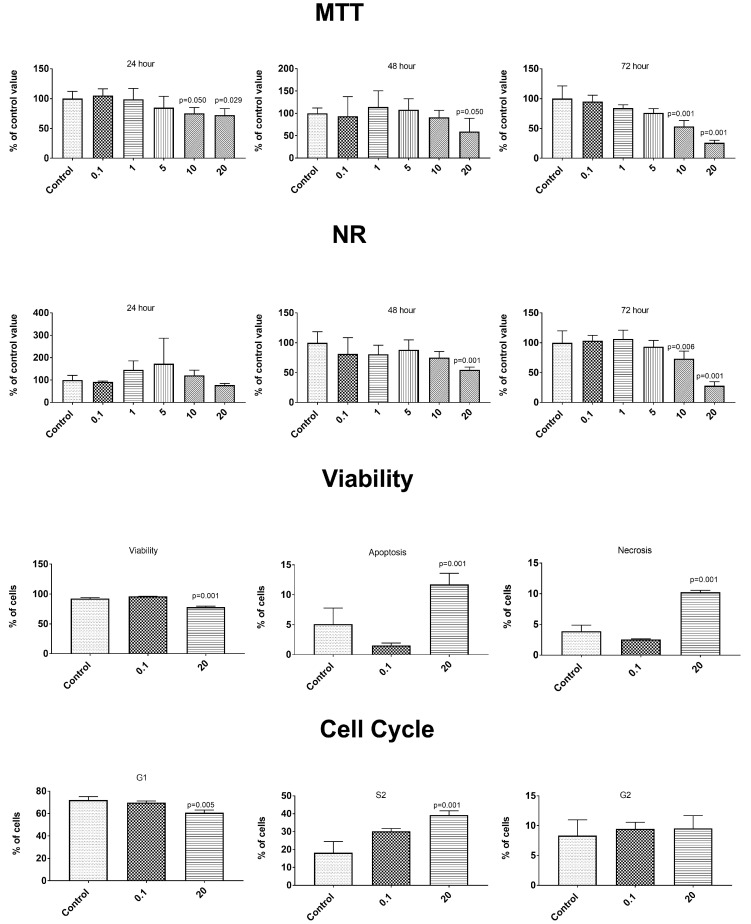
Impact of JA-GD (sample after the in vitro digestion of the JA-elicited lovage leaves) on cytotoxicity (MTT and NR method), type of cell death (Annex V and PI test), and phases of the cell cycle in the HPrEC ATCC^®^ PCS-440-010™ cell line.

**Figure 5:**
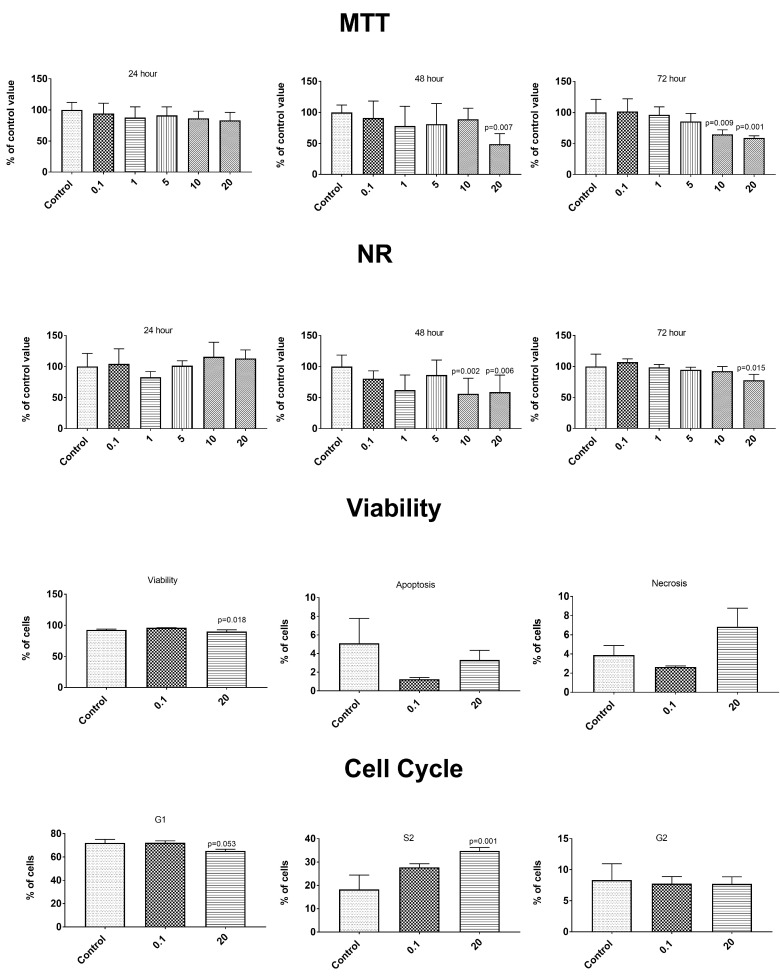
Impact of YE-GD (sample after the in vitro digestion of the YE-elicited lovage leaves) on cytotoxicity (MTT and NR method), type of cell death (Annex V and PI test), and phases of the cell cycle in the HPrEC ATCC^®^ PCS-440-010™ cell line.

The anticancer properties were determined using two cancer lines—gastric epithelial cancer cell line NCI-N87 (ATCC^®^ CRL5822™) and prostate epithelial cancer line VCaP—ATCC^®^ CRL-2876™. After the exposure to the tested concentration of the PBS extracts from the control and elicited lovage, no changes were observed in the cancer cell metabolism or in the control epithelial cells of the healthy prostate line HPrEC ATCC^®^ PCS-440-010™ (SUPPLEMENT—Figures S1–S15). The simulated gastrointestinal digestion induced a release of some compounds influencing the metabolism of the control cells (Figure 3, Figure 4 and Figure 5). A concentration-dependent decrease in the cell number in both tests (MTT and NR) was observed throughout the incubation time. Statistically significantly higher cytotoxic changes were noted after 72 h of incubation with the extracts at a concentration of 10 and 20 mg/mL. The changes in the number of cells were caused by an enhanced process of apoptosis (extracts at the concentration of 20 mg/mL) and changes in the cell cycle (a decrease in the percentage of G1 phase cells and an increase in the percentage of S phase cells—extracts at the concentration of 20 mg/mL)—Figure 3, Figure 4 and Figure 5. We observed differences in the effects caused by the in vitro digested samples, i.e., the control samples (C-GD), JA-elicited lovage leaves (JA-GD), or YE-elicited lovage leaves (YE-GD). Generally, the addition of C-GD and JA-GD exerted a similar effect on the cell count measured by the MTT and NR assays (lower at the concentration of 10 and 20 mg after the 72-h treatment), influenced the viability of the cells (a decreased level of viable cells and an increased level of apoptotic and necrotic cells), and contributed to an increase in the number of S-phase cells. The in vitro digested samples from the YE-elicited lovage leaves (YE-GD) did not exhibit any cytotoxicity. There was also a reduction of the cell count measured by the MTT and NR assays (not as high as in the control or JA-GD samples) and an increase in the percentage of cells in the S phase; however, the sample did not induce apoptosis and necrosis at the 20 mg/mL concentration. These findings indicate that the fresh Levisticum officinale leaves elicited with YE are safe for the healthy prostate line even at high concentrations.

## 4. Discussion

Polyphenols are a very important group of bioactive compounds contained in herbs, including lovage [1]. These secondary metabolites have attracted considerable interest and attention due to their multiple bioactivities. In plants, this group of metabolites serves an important function in growth, reproduction, and induced plant resistance. Therefore, their production can be induced in response to stress conditions or attacks by some pathogens, predators, or parasites [36]. There are many investigations of the effect of some methods imitating signal transduction pathways and inducing plant resistance via the production of phytochemicals, e.g., polyphenols, by plants [13,28]. As a method of induction of plant resistance and secondary metabolism, elicitation is part of the research trends aimed at increasing the value of health-enhancing food of plant origin by inducing the biosynthesis of phytochemical compounds, including polyphenols [37,38,39].

The overproduction of phenolic compounds induced by elicitation has been documented in many herbs and vegetables [14,20,39], but there is still limited information about the potential bioavailability of phenolic compounds from elicited vegetables or herbs in the literature. Recently, it has been observed that scientists recognize the importance of this issue and there are several examples of research on this topic, such as a study conducted by Świeca [17] on phenolic compounds from elicited buckwheat sprouts, which were characterized by a high bioavailability, correlated with high antioxidant activities. Similarly, Złotek et al. [18] demonstrated the high bioavailability of phenolic compounds from basil elicited with arachidonic acid.

As shown in Table 1, seven phenolic acids (protocatechuic acid, hydroxybenzoic acid, siringic acid, vanillic acid, sinapic acid, salicylic acid, and caffeic acid) were identified in the PBS samples and in the GD samples (with the exception of vanillic acid and caffeic acid in the latter samples). Similar differences in the content of some phenolic compounds were observed in the samples before and after the in vitro digestion in a study conducted by Złotek et al. [18]. The elicitation (mainly with jasmonic acid) caused an increase in most of the identified phenolic acids in the PBS extracts, but this effect was noted in the potentially available fractions of phenolic compounds (GD samples) only in the case of protocatechuic acid and hydroxybenzoic acid—Table 1. There are reports confirming the effect of jasmonic acid and yeast extract on the induction of phenolic compound biosynthesis. For instance, JA-elicitation increased luteolin, ferulic, caffeic, chlorogenic, and *o*-coumaric acid levels in lettuce [39], elicitation with JA increased the content of rosmarinic acid and benzoic acid in basil leaves [19], and YE-elicitation yielded a statistically significant increase in the ferulic, chicoric, and caffeic acid levels in lettuce [20]. However, these studies are predominantly focused on “chemical extracts”.

It is well documented that phenolic compounds exhibit many biological activities, e.g., antioxidant, anti-inflammatory, anticarcinogenic, antidiabetic, antimicrobial, or gastroprotective effects, which are valuable in the prevention of some chronic or degenerative diseases. The bioaccessibility and biological activity in an in vitro digested sample may provide detailed information about the actual impact of the compounds contained in plant material on their bioactivity in the organism. During digestion in the gastrointestinal tract, some phenolics can be released from the food matrix, whereas others can be complexed with digesting enzymes and some metabolites can be formed from phenolic compounds whose biological activity may differ from that of the initial materials [40,41]. For this reason, the present study determined the broad-sense biological activity of extracts from fresh lovage leaves elicited with JA and YE in pre-digested (PBS) and digested (GD) samples.

Surprisingly, the analysis of the content of phenolic acids in the PBS and GD-extracts revealed that the in vitro digestion caused an increase only in the content of hydroxybenzoic acid (Table 1). Similarly, in a study conducted by Ng et al. [42], in vitro digestion in general reduced the content of phenolic compounds in a majority of functional plant foods. However, another study reported that simulated digestion resulted in an increase in the phenolic content in edible plants and vegetable juices [43,44]. As suggested by the authors mentioned above, the effect of in vitro digestion on the content of phenolic compounds depends on several factors, e.g., the plant matrix, plant species, extraction solvents, or digestion model [42]. As in the present study, Wong et al. [45] showed that phenolic acids were sensitive to the intestinal digestion conditions, and their results suggested that a large proportion of these compounds could be transformed into other unknown or undetected structural forms with different properties.

Hydroxybenzoic acid (whose content increased in the present study after the in vitro digestion) and its derivatives exhibit many biological properties, e.g., antioxidant, anti-inflammatory, antimicrobial, antimutagenic, hypoglycemic, and cardioprotective activities confirmed by many researchers [46,47]. For example, Su et al. [48] reported significant correlations between the content of phenolic acids and the antioxidant and anti-inflammatory potential of blueberry extracts.

In the previous research, the simulated digestion of control and arachidonic acid-elicited basil samples resulted in an increase in some biological activities, e.g., antioxidant, anti-inflammatory, antidiabetic, and anticancer properties [23]. These observations were partially confirmed in the present study, as the in vitro digestion significantly improved some biological activity in the lovage leaves (control and elicited), in particular, the ability to inhibit the activity of pro-inflammatory enzymes and those involved in the pathogenesis of the metabolic syndrome. Additionally, antimicrobial properties and some of the tested antioxidant activities (antiradical and reducing power abilities) were improved—Table 2 and Table 3, Figure 1. The reducing power potential was also reported to increase after in vitro digestion in the case of four plants. i.e., *Ginkgo biloba, Lycium barbarum, Clitoria ternatea,* and *Dioscorea polystachya* in a study performed by Ng et al. [42], whereas an opposite effect was observed in the same publication for other plants (*Cymbopogon citratus, Morus alba, Centella asiatica*, and *Angelica sinensis*). Other studies showed that the antiradical potential in some plants and vegetable juices was relatively stable after in vitro digestion [43].

Inflammation is part of the complex biological response to injury, infection, or some stress conditions, but it is also a central feature of many pathological conditions. It may lead to many diseases such as atherosclerosis, obesity, metabolic syndrome, diabetes, and some types of cancers. In addition to the inflammatory mediators such as interleukins, tumor necrosis factor, nuclear factor-κB, intercellular adhesion molecule-1, prostaglandin E2, and inducible nitric oxide synthase, there are also inducible cyclooxygenase-2 (COX-2) and 5-lipoxygenase (LOX) whose overproduction or increased activity leads to different types of cell damage [49]. The LOX enzymes play a key pro-inflammatory role through the metabolism of arachidonic and linoleic acids to leukotrienes. COX2 levels, in contrast to the constitutive form COX1, are dramatically up-regulated in inflamed tissues. Therefore, a great deal of interest has been focused on the co-inhibition of both COX2 and LOX pathways to address inflammatory disorders while minimizing the side effects [50]. One of the mechanisms of the anti-inflammatory action of phenolic compounds proposed in the literature is inhibition of the synthesis of pro-inflammatory mediators, e.g., by inhibiting the activity of the lipoxygenase and cyclooxygenase 2 enzymes [11]. In the present study, the potentially bioavailable phenolic acid fractions from the control and elicited lovage exhibited high potential to inhibit LOX and COX2 activities (Figure 1). The significant increase in LOX and COX2 inhibition after the simulated digestion process observed in the present study (Figure 1) may be connected with the increased content of hydroxybenzoic acid in the GD samples (Table 1), since this compound is very effective in suppressing inflammation and cell proliferation and in promoting apoptosis, as indicated in some studies [51]. Additionally, as reported in some literature, digestion in the gastrointestinal tract may be accompanied by the formation of some metabolites from phenolic compounds whose biological activity may differ from that of the initial materials, and these metabolites are not identified [40,41], which may be the cause of the increase in some biological activity of GD samples.

There are many reports of the effects of phenolic compounds on cancer cell lines [52,53,54]. Unfortunately, the present study did not show any changes in the cancer cell metabolism after the exposure to the experimental concentrations of extracts from the control and elicited lovage (Figures S1–S15). Additionally, there are limited numbers of studies on the cytotoxity of phenolic compounds/plant extracts on healthy cells. Some studies have indicated that some phenolic compounds (or other bioactive compounds contained in plant extracts) may act as antioxidants within a certain dose range, because the same compounds at high doses may have a pro-oxidative effect [54]. For example, eugenol has antioxidant and anti-inflammatory properties at low concentrations but may act as a pro-oxidant at higher concentrations [55]. Additionally, in a study conducted by Bacanli et al. [54], some phenolics decreased the cell viability of healthy cells V79 with an increasing dose. These observations suggest the importance of investigations of the effect of dietary bioactive compounds on healthy cell lines, conducted to check the safety of doses consumed in the diet. In the present study, the samples subjected to simulated gastrointestinal digestion caused a decrease in the number of cells of the healthy prostate line HPrEC ATCC^®^ PCS-440-010™, but only at the highest concentrations studied (10 mg/mL and 20 mg/mL)—Figure 3, Figure 4 and Figure 5. It should be noted that most of the biological properties (especially antioxidant and anti-inflammatory activities) of these samples relate to the lower concentrations—Figure 1 and Table 2. Additionally, the elicitation of lovage with the yeast extracts substantially reduced the effect on the metabolism of healthy cells, compared to the control lovage samples (Figure 3, Figure 4 and Figure 5).

The use of food compounds in prevention of human diseases has been a focus of considerable public and scientific interest in recent years. New food products may be a good source of ingredients with high biological potential such as an inhibitory effect on civilization diseases, e.g., cancers or metabolic syndrome (MS). MS comprises obesity, insulin resistance, hypertension, disorders in glucose metabolism, and dyslipidemia manifested by elevated triglyceride levels and low HDL concentrations. All of these factors are the main causes of development of atherosclerosis; hence, MS is described as a significant risk of coronary heart disease [56]. Due to their biological activity, phenolic compounds play a special role in the prevention of diseases and the maintenance of good health, especially the antioxidant and anti-inflammatory activities and the inhibition of enzymes involved in MS pathogenesis (syringic acid). Among polyphenols, phenolic acids are the major group of biologically active compounds with therapeutic activities in many diseases, e.g., cardiovascular disease [54]. In this study, the angiotensin converting enzyme (ACE) inhibitory activity of elicited lovage leaves was determined in the buffer samples (PBS) and gastrointestinally digested (GD) extracts. ACE is one of the main enzymes in the rennin–angiotensin–aldosterone system, which contributes to the regulation of arterial blood pressure and water and electrolytic balance [57]. ACE catalyzes the production of a potent vasoconstrictor octapeptide (angiotensin II) from inactive decapeptide (angiotensin I) and, in parallel, the breakdown of bradykinin, i.e., a vasodilator peptide. Synthetic ACE inhibitors are commonly used as drugs in the treatment of hypertension, but they may cause serious side effects such as cough, rash, or angioedema [58]. Natural ACE inhibitors, which can support hypertension treatment, represent various chemical groups, such as peptides [28], polyphenols [59], or carotenoids [60]. As shown in Table 3, all of the tested extracts were characterized by ACE inhibitor activity. It should be noted that the gastrointestinal digestion enhanced this property. The analyzed phenolic compounds include phenolic acids with documented ACE inhibitory potential. As shown by Błaszczak et al. [59], pure caffeic acid is characterized by an IC_50_ value of 747.22 µg/mL. The contribution of hydroxyl, carboxyl, and acrylic acid groups in the structure of phenolic acids is the main factor of their ability to inhibit ACE [61]. Moreover, hydroxybenzoic acids have the potential to ameliorate cardiovascular problems related to aging, such as hypertension, atherosclerosis, and dyslipidemia [62]. It should be noted that syringic acid as a compound of lovage leaf extracts showed anti-hypertensive activity in induced hypertensive rats [63]. The results indicated that the oral administration of 50 mg/kg b.w. of syringic acid in rats had anti-hypertensive activity, decreased lipid peroxidation, and increased nitric oxide availability and levels of antioxidants in rat blood samples.

It is worth noting that the mortality and morbidity in cardiovascular diseases have been shown to be elevated in overweight individuals, particularly with a central deposition of adipose tissues, which develops due to an imbalance between energy consumption and expenditure in the organism. The mechanism of action of food compounds against obesity is multidirectional but is still not elucidated. The main actions include the reduction of dietary-fat absorption, energy intake, and adipogenesis and lipogenesis processes as well as the suppression of appetite or an increase in energy expenditure and lipolysis. One of the most important and promising approaches to reduce the energy intake through gastrointestinal mechanisms is inhibition of enzymes hydrolyzing the main high-energy compounds in food. The main enzyme that hydrolyzes triacyclglycerols is pancreatic lipase produced by pancreatic acinar cells. It splits fats into absorbable monoacylglycerols and fatty acids. This enzyme is responsible for the hydrolysis of 50–70% of total dietary fats in the gastrointestinal tract [61,62,63].

Polyphenol-rich extracts from elicited lovage leaves are effective inhibitors of pancreatic lipase in vitro (Table 3). There are several studies of polyphenols as pancreatic lipase inhibitors. Some results indicated that increasing phenolic hydroxyl groups in polyphenols increased their binding affinities and inhibition of lipase [64].

The antidiabetic potential of phenolic compounds is widely described, but the mechanisms of the action of these phytochemicals in the diabetic disorder are still being discussed. The elucidation of these mechanisms is complicated due to the large number of phenolic phytochemicals and their potential bioavailability and biotransformation in the human organism. One of the proposed mechanisms is associated with the potential of phenolic compounds to inhibit α-amylase and α-glucosidase, i.e., two key enzymes required for starch digestion in humans [5,10,65]. The inhibition of these enzymes can retard carbohydrate digestion, thus causing a reduction in the rate of glucose absorption into the blood [66,67]. Moreover, type I diabetes is also caused by the progressive destruction of pancreatic insulin-producing β cells, which can be damaged by lipids entering and accumulating in this organ. Hence, pancreatic lipase inhibitors may have protected the pancreas against damage and contributed to the production of normal levels of insulin [67]. It should be noted that caffeic and syringic acids have been described as good enzyme inhibitors involved in carbohydrate metabolism [68]. Moreover, protocatechuic acid is described as an α-amylase inhibitor with an IC_50_ value of 12.5 μmol/L [69]. Other study results demonstrated α-glucosidase and α-amylase enzyme inhibitory activities in extracts of *Elaeagnus angustifolia* leaves, which may be attributed to the presence of vanillic acid and 4-hydroxybenzoic acid, thereby confirming its traditional use for the management of diabetes mellitus [68]. It should be noted that these compounds were identified in the extracts tested in the present study as well (Table 1).

Foodborne pathogens are responsible for foodborne infections with considerable effects on human health [69]. Most food-poisoning reports are associated with bacterial contamination, especially by representatives of Gram-negative bacteria such as *Salmonella typhi* or *Escherichia coli* and Gram-positive bacteria: *Staphylococcus aureus, Listeria monocytogenes*, and *Bacillus cereus* [70]. Yeast *C. albicans* are commensal microorganisms growing on the skin and mucosal surfaces of healthy individuals [71]. However, in some cases, they are associated with opportunistic infection in humans, especially in immunocompromised patients, such as those with HIV/AIDS [70,71]. There is an increasing number of studies showing microbial resistance to chemical agents e.g., antibiotics. Therefore, effective, healthy, safe, and natural antimicrobials are still being sought. Recent studies show that plant extracts have antimicrobial properties [69,72,73,74]. Due to the increase in the resistance to conventional antibiotics and the trend associated with the avoidance of synthetic preservatives, the antimicrobial properties of polyphenols are highly promising. The antimicrobial activity of polyphenols occurring in vegetable foods and mushrooms have been extensively investigated against a wide range of microorganisms e.g., foodborne pathogens [75,76]. Polyphenols have high antimicrobial activity and are able to suppress a number of microbials (by the reduction of host ligand adhesion and the neutralization of bacterial toxins). A previous study indicated that dihydroxybenzoic and protocatechuic acids exhibited high antimicrobial activity against Gram positive bacteria *S. aureus* and *L. monocytogenes* and yeast *C. albicans* [76,77]. Antifungal activity of syringic and sinapic acids against *C. albicans* was detected as well [77]. Mekinić et al. [78] detected the antibacterial activity of common yarrow extracts with a high content of hydroxybenzoic acid derivatives against *B. cereus*. The present study indicated a high antimicrobial activity of the lovage leave extract. This is in agreement with a previous study [3]. Spréa et al. [3] demonstrated the antibacterial activity of lovage extracts towards *E.coli, S. aureus*, and *L. monocytogenes* with MIC values between 5 and 10 mg/mL, 2.5 and 10 mg/mL, and 2.5 and 5 mg/mL, respectively. At the same time, the authors showed the bactericidal activity of the tested lovage extract above 20 mg/mL [3]. It should be emphasized that lovage extracts are a source of substances that inhibit the growth of pathogenic bacteria responsible for food poisoning. Previous literature has suggested that such medicinal plants as lovage may provide a promising approach as adjuvant therapy, since several studies have demonstrated that antibiotic activity can be potentiated in combination with phytochemicals [3].

The research presented in this paper confirms these reports. In terms of the impact on human health, particularly important are the properties observed in the in vitro digestion tests, while samples before the simulated digestion process did not show antimicrobial properties (Table 4). This suggests a possibility of their use in the prevention or supportive treatment of food poisoning (rather than in food preservation).

## 5. Conclusions

Summarizing, the present results indicate that the JA elicitation resulted in a higher increase in the phenolic acid biosynthesis in the PBS extracts. The JA elicitation resulted in a higher increase in the phenolic acid biosynthesis; hence, this elicitation technique can be used in industrial application for the production of aromatic acids that can be applied as anti-inflammatory and antioxidant agents. However, the analysis of the bioactive properties demonstrated that the fresh lovage samples elicited with the yeast extract (especially after the in vitro digestion) showed promising health-promoting properties and were safer (also at the high doses) to the healthy prostate cell line. These results are particularly important, because yeast extract can be used in various plant production systems as a natural, environmentally friendly, and safe elicitor improving the health-promoting qualities of lovage leaves in the human digestive tract, as confirmed in the simulated digestion conditions in this study. Additionally, as an edible plant, lovage can be cultivated in homes using yeast extract elicitation, which is an additional advantage of this elicitor.

## Figures and Tables

**Table 1:** Qualitative and quantitative analysis of the phenolic acids in the control, elicited lovage buffer (PBS (phosphate buffered saline)) extracts, and gastrointestinally digested (GD) extracts. C-PBS—PBS extracts from the control plants; JA-PBS—PBS extracts from the plants elicited with the solution of 10 µM jasmonic acid; YE-PBS—PBS extracts from the plants elicited with the 0.1% yeast extract: C-GD—gastrointestinally digested extracts from the control plants; JA-GD—gastrointestinally digested extracts from the plants elicited with the solution of 10 µM jasmonic acid; YE-GD—gastrointestinally digested extracts from the plants elicited with the 0.1% yeast extract; nd—not detected. Values in the rows designated by the different letters are significantly different (*p* < 0.05).

Compounds (µg/g FW)	Sample					
	C-PBS	C-GD	JA-PBS	JA-GD	YE-PBS	YE-GD
Protocatechuic acid	6.63 ± 0.92a	4.85 ± 0.00a	10.52 ± 0.08b	5.23 ± 0.37a	nd	4.33 ± 0.02a
Hydroxybenzoic acid	3.56 ± 0.04a	21.88 ± 0.00b	3.74 ± 0.01a	35.21 ± 4.04c	3.06 ± 0.00a	35.75 ± 1.65c
Siringic acid	4.23 ± 0.28a	4.15 ± 1.19a	10.53 ± 0.04b	4.47±0.88a	nd	3.33 ± 0.03a
Vanillic acid	5.54 ± 0.37a	nd	10.10 ± 0.01b	nd	5.58 ± 1.83a	nd
Sinapic acid	18.78 ± 0.62b	9.28 ± 1.82a	40.48 ± 0.03d	8.09 ± 1.51a	33.80 ± 0.13c	9.74 ± 0.25a
Salicylic acid	9.59 ± 1.72a	nd	52.57 ± 0.07b	11.32 ± 6.01a	nd	nd
Caffeic acid	5.80 ± 0.56a	nd	18.16 ± 0.02b	nd	nd	nd

**Table 2:** Antioxidative activity in the control, elicited lovage buffer (PBS) extracts, and gastrointestinally digested (GD) extracts. C-PBS—PBS extracts from the control plants; JA-PBS—PBS extracts from the plants elicited with a solution of 10 µM jasmonic acid; YE-PBS—PBS extracts from the plants elicited with 0.1% yeast extract; C-GD—gastrointestinally digested extracts from the control plants; JA-GD—gastrointestinally digested extracts from the plants elicited with a solution of 10 µM jasmonic acid; YE-GD—gastrointestinally digested extracts from the plants elicited with 0.1% yeast extract; ABTS—radical scavenging ability against ABTS; RP—reducing power; CHP—chelating power. EDTA—Ethylenediaminetetraacetic acid. Values in the rows designated by the different letters are significantly different (*p* < 0.05).

Antioxidant Activity	Sample					
	C-PBS	C-GD	JA-PBS	JA-GD	YE-PBS	YE-GD
ABTS (µM Trolox/g FW)	3.20 ± 0.66a	10.40 ± 1.34d	4.60 ± 0.78ab	7.27 ± 1.77c	3.05 ± 0.43a	6.49 ± 1.28bc
RP (mg Trolox/g FW)	10.02 ± 2.01ab	8.86 ± 1.07ab	8.16 ± 1.23a	12.87 ± 2.44b	7.76 ± 1.13a	11.21 ± 2.90ab
CHP (mg EDTA/g FW)	711.71 ± 13.91b	572.44 ± 73.86a	725.62 ± 4.64b	575.61 ± 8.95a	687.37 ± 48.98b	572.44 ± 15.92a

**Table 3:** ACE, lipase, α-amylase, and α-glucosidase inhibitory activity in the control, elicited lovage buffer (PBS) extracts, and gastrointestinally digested (GD) extracts. C-PBS—PBS extracts from the control plants; JA-PBS—PBS extracts from the plants elicited with a solution of 10 µM jasmonic acid; YE-PBS—PBS extracts from the plants elicited with 0.1% yeast extract; C-GD—gastrointestinally digested extracts from the control plants; JA-GD—gastrointestinally digested extracts from the plants elicited with a solution of 10 µM jasmonic acid; YE-GD—gastrointestinally digested extracts from the plants elicited with 0.1% yeast extract; ACE—angiotensin converting enzyme; EC_50_—an extract concentration (mg FW/mL) providing 50% inhibition; na—no activity. Values in the rows designated by the different letters are significantly different (*p* < 0.05).

EC_50_ (mg FW/mL)	Sample					
	C-PBS	C-GD	JA-PBS	JA-GD	YE-PBS	YE-GD
ACE	199.93 ± 5.65d	8.19 ± 0.78a	102.49 ± 4.78c	33.88 ± 1.10 b	97.68 ± 8.83c	24.72 ± 1.72 b
lipase	2.14 ± 0.15e	0.27 ± 0.02a	1.15 ± 0.05c	0.78 ± 0.06b	1.85 ± 0.13c	0.79 ± 0.08 b
α-amylase	na	20.82 ± 1.07b	na	33.88 ± 1.49c	na	17.63 ± 0.81a
α-glucosidase	13.11 ± 1.85b	2.64 ± 0.16a	24.18 ± 1.54d	3.10 ± 0.73a	19.67 ± 1.02c	2.52 ± 0.29a

**Table 4:** Antimicrobial activity of the control, elicited lovage buffer (PBS) extracts, and gastrointestinally digested (GD) extracts against bacteria and yeast. C-PBS—PBS extracts from the control plants; JA-PBS—PBS extracts from the plants elicited with a solution of 10 µM jasmonic acid; YE-PBS—PBS extracts from the plants elicited with 0.1% yeast extract; C-GD—gastrointestinally digested extracts from the control plants; JA-GD—gastrointestinally digested extracts from the plants elicited with a solution of 10 µM jasmonic acid; YE-GD—gastrointestinally digested extracts from the plants elicited with a 0.1% yeast extract; MIC—minimum inhibitory concentration; MLC—minimum lethal concentrations; nd—not detected; ns—not studied. ATCC—American Type Culture Collection.

Antimicrobial Activity	Sample								
	*C-PBS*	*C-GD*	*JA-PBS*	*JA-GD*	*YE-PBS*	*YE-GD*	Penicilin G ***	Streptomycin ***	Cyclohexamide ***
**E. coli* ATCC 25922*	Nd */nd **	2.5 */>5.0 **	Nd */nd **	5.0 */>5.0 **	Nd */nd **	2.5 */>5.0 **	7.81/7.81	15.62/15.62	ns
*S. aureus ATCC 29737*	Nd */nd **	5.0 */>5.0 **	Nd */nd **	5.0 */>5.0 **	Nd */nd ***	5.0 */>5.0 **	>250/>250	7.81/7.81	ns
*S. enterica ATCC 4931*	Nd */nd **	5.0 */>5.0 **	Nd */nd **	5.0 */>5.0 **	Nd */nd **	5.0 */>5.0 **	7.81/7.81	3.90/3.90	ns
*B. cereus ATCC 14579*	Nd */nd **	1.25 */>2.5 **	Nd */nd **	2.5 */5.0 **	Nd */nd **	1.25 */2.5 **	>250/>250	15.62/15.62	ns
*L. monocytogenes ATCC BAA-2660*	Nd */nd **	5.0 */>5.0 **	Nd */nd **	5.0 */>5.0 **	Nd */nd **	5.0 */>5.0 **	1.95/1.95	3.90/3.90	ns
*C. albicans ATCC 90028*	Nd */nd **	1.25 */2.5 **	Nd */nd **	2.5 */5.0 ***	Nd */nd **	2.5 */5.0 **	ns	ns	15.62/15.62

* Minimum inhibitory concentration MIC (mg FW mL^−1^), ** Minimum lethal concentrations (MLC) (mg FW mL^−1^), *** (µg mL^−1^).

## Supplementary Materials

The supplementary materials are available online at https://www.mdpi.com/2076-3921/9/6/554/s1.
